# The Impact of Heat Load on Cattle

**DOI:** 10.3390/ani9060322

**Published:** 2019-06-06

**Authors:** Angela M. Lees, Veerasamy Sejian, Andrea L. Wallage, Cameron C. Steel, Terry L. Mader, Jarrod C. Lees, John B. Gaughan

**Affiliations:** 1 School of Agriculture and Food Sciences, The University of Queensland; Gatton, QLD 4343, Australia; a.wallage@uq.edu.au; 2 School of Environmental and Rural Science, University of New England, Armidale, NSW 2350, Australia; csteel5@une.edu.au (C.C.S.); Jarrod.Lees@une.edu.au (J.C.L.); 3 Indian Council of Agricultural Research (ICAR)-National Institute of Animal Nutrition and Physiology, Adugodi, Bangalore 560030, India; drsejian@gmail.com; 4 Department of Animal Science, University of Nebraska, Lincoln, NE 68588, USA; tmader12@msn.com; 5 Mader Consulting, Gretna, NE 68028, USA

**Keywords:** cattle welfare, climate change, heat load, heat stress, mitigation techniques, multiple stressors, production, reproduction, thermotolerance

## Abstract

**Simple Summary:**

It is well known that the thermal environment has an integral role in maintaining the health and productivity of cattle. Although cold stress has been identified to negatively influence cattle comfort and productivity, the predominant focus herein has been describing the influence of heat stress on bovines. The impact of heat stress is particularly important due to the changing global environment. Global warming is likely to occur, however, the nature and magnitude of environmental changes, both climatic and non-climatic, are difficult to elucidate. Therefore a predominant focus on the impact of hot environments on cattle is warranted. This review provides an overview of the dynamic relationship that exists between the thermal environment and bovines.

**Abstract:**

Heat stress and cold stress have a negative influence on cattle welfare and productivity. There have been some studies investigating the influence of cold stress on cattle, however the emphasis within this review is the influence of heat stress on cattle. The impact of hot weather on cattle is of increasing importance due to the changing global environment. Heat stress is a worldwide phenomenon that is associated with reduced animal productivity and welfare, particularly during the summer months. Animal responses to their thermal environment are extremely varied, however, it is clear that the thermal environment influences the health, productivity, and welfare of cattle. Whilst knowledge continues to be developed, managing livestock to reduce the negative impact of hot climatic conditions remains somewhat challenging. This review provides an overview of the impact of heat stress on production and reproduction in bovines.

## 1. Introduction

The thermal environment can have a negative influence on cattle welfare. Historically, Ames [1] defined the thermoneutral zone as the thermal environment where an animal experiences optimum health and maximum productivity. Whilst cattle comfort and productivity may be compromised during exposure to cold, wet and/or windy conditions [2,3], there has been a predominant focus on the influence of hot weather on cattle, and other species. The impact of hot weather on cattle is of increasing importance, particularly in conjunction with the changing global environment.

Beyond the direct impact that heat stress has on the health and productivity of animals, the economic impact on livestock producers also needs to be considered. In 2003, St-Pierre et al. [4] estimated that heat stress had an annual economic burden of between $1.69 and $2.36 billion (USD) on the US animal agriculture industries. Within this estimate, economic losses of $897 to $1500 million (USD) were attributed to the dairy industry and $370 million (USD) for the beef industry [4]. Sackett et al. [5] estimated the economic costs of heat stress to Australian feedlots at approximately 16.5 million (AUD). Given that these analyses were conducted over a decade ago, these estimates may not reflect the current economic impact of heat stress. Furthermore, in conjunction with climate change, it is probable that these estimates are underestimating the economic impact of heat stress on cattle production systems.

For livestock production enterprises, climate change has the potential to alter the thermal environment, which may result in the climate having an increasingly negative impact on the welfare and productivity of cattle. Periods of hot weather are already associated with reduced animal health, reduced reproductive efficiency in both males and females, and decreased feed conversion efficiency [4,6]. Therefore, it is likely that climate change will have a considerable impact on the economic viability of animal agriculture worldwide.

In spite of this, all animals possess the capacity to adapt to their thermal environment. Animals are capable of modifying their behavioral, physiological, and morphological characteristics, or a combination of these, in response to the thermal environment [7]. This review has attempted to provide a rounded overview of the impact that heat stress has on bovines. 

## 2. Climate Change

The effect of climate change is highly variable globally and is largely influenced by geographical location. Cattle and livestock enterprises have the ability to adapt to an increasing mean global temperature, the primary concern, however, is the ability of livestock to cope with climatic extremes, e.g., heat waves [8]. Climate change has the potential to present as (i) rapid changes in climate over a couple of years or (ii) as more subtle changes over decades [8]. However, irrespective of the manifestation of climate change, global warming is likely to have a significant impact on the stability and sustainability of livestock production worldwide. Globally, various climate change models are predicting a 1.1 °C to 6.4 °C increase in temperature by the end of this century [9]. Furthermore, in southern Australia, the average number of consecutive days of heat-stress has increased from two days per heat stress event from 1960 to 1999, to four days from 2000 to 2008 [10].

Numerous species are likely to be negatively impacted by the changing global environment [11], due to changes in ecosystem microclimates. Many species have adaptations to cope with short-term climate variability, i.e., seasonal changes. However, these adaptations may not be successful for species survival with the predicted climate change [11]. Predicting the effect of climate change on livestock is somewhat challenging due to the interrelationships that exist between the animal and its surrounding environment, and the impact of human activity on these relationships [8]. It is also important to consider the indirect effects of climate change on soil fertility and degradation, water availability, grain yield, quality and availability, and spread of diseases/pathogens that may potentially impact the cattle producers and their ability to manage periods of hot weather [9,12]. 

Irrespective of livestock productions contribution to climate change, animal production needs to increase to satisfy consumer demand. A challenge regarding the effects of climate change on livestock enterprises is how dependent the enterprise is on the thermal environment and what can be implemented to offset the impacts of increasing temperatures [9]. The current effect of the thermal environment is estimated by the impact of climatic conditions on animal performance, health, and welfare [9]. 

## 3. Heat Wave Events

Heat waves are defined as a number of successive days, typically three to five, where maximum ambient conditions are above a specific threshold [13,14]. One predicted consequence of climate change is the increased prevalence and intensity of heat waves [15]. Climatic trends of heat waves differ from summer to summer, and future predictions suggest that the climatic behavior of heat wave events over the years will continue to be varied [16,17]. Gaughan and Cawdell-Smith [8] suggested that there is little doubt that there has been an increase in heat waves since the 1990’s. Although, over the last 50 years there has been a significant advancement in the ability to predict and forecast climatic events [16]. This ability to forecast heat wave events has enabled livestock producers to implement mitigation strategies to prepare for forthcoming adverse climatic events. 

The effects of heat waves on individual cattle are influenced by the intensity and duration of the heat wave. It is well documented that feedlot cattle can be particularly susceptible to changes in climatic conditions [18,19,20]. The susceptibility of feedlot cattle to heat load has been emphasized during prolonged heat wave events and where conditions manifest with limited nighttime relief [18,20]. Numerous authors have reported heat wave conditions where cattle, particularly feedlot cattle, have succumbed to heat load, for example:

- February 1991–4000 deaths were recorded in Queensland (Australia) [21], with one feedlot reporting 2680 deaths [22] during a heat wave event with high relative humidity and limited air movement
- July 1995–3750 deaths were estimated in Western Iowa, [23], and total deaths for the mid-central US were over 4000 cattle [24]. This particular heat wave was associated with an estimated economic loss of approximately $28 million contributed from production losses [20]
- Hahn [20] reported the loss of 100 feedlot cattle in central Nebraska over a heat wave that had three spikes in thermal loads. Deaths occurred during the third spike where it was hypothesized that ad libitum feed intake resulted in large metabolic heat load and in conjunction with environmental heat load, surpassed the animals’ ability to maintain thermal balance [20]
- 1999–over 5000 feedlot cattle died during an extreme heat wave in north-eastern Nebraska [25,26]
- February 2000–1255 cattle died in southwestern New South Wales with deaths occurring after a rainfall event where climatic conditions presented high relative humidity and high overnight ambient temperature [22]
- June 2017–4000 to 6000 dairy cows died in Fresno, Kings and Tulare counties USA [27] during a heat wave

## 4. Defining Heat Load

Traditionally, the impact of hot weather has been referred to as heat stress. Buffington et al. [28] suggested that heat stress is caused by a combination of environmental conditions that result in the effective temperature of the environment to be greater than the temperature range of the thermoneutral zone. This is somewhat misleading as the term heat stress by definition refers to the combination of environmental conditions alone without consideration of animal factors [21,28]. However, factors, such as genotype, coat type and coat color, diet type and diet composition, body condition, i.e., fat coverage and deposition, performance, i.e., growth and lactation, health status, and degree of adaptation, are known to influence thermal balance. Thus, throughout this review, the term heat load will be used rather than heat stress, as the term heat load incorporates the cumulative effects of animal factors and environmental conditions on the thermal comfort of animals [21] and, therefore, becomes a better descriptor of an animal’s thermal balance.

### Multiple Stressors

Animals that are adapted to a hot climate generally exhibit reduced growth and reproductive efficiency [29], which is associated with the adaptive mechanisms that ensure survival [30]. In extensive grazing systems, it has been identified that climatic constraints are not the only factor that negatively influences livestock production. The indirect effects of climate change will also influence pasture resources [31], potentially depriving grazing animals of nutrient requirements. Similarly, the changing climate may also result in droughts, ultimately resulting in feed and water scarcity for grazing animals. These situations can be associated with a decrease in growth and reproductive efficiency in livestock [32]. Furthermore, these animals may also be required to walk long distances under high solar loads to find feed and water, imposing locomotor stress on grazing animals [33]. Therefore, it is important to consider the impact of multiple stressors on livestock, this is particularly important to consider in conjunction with climate change, as it is unlikely that animals will be exposed to a single stressor. 

Numerous sheep and goat studies have evaluated the impact of multiple environmental stressors (heat, nutritional, and walking) on production, reproduction, and ability to cope with stressful conditions [32,34,35,36,37]. These studies have identified that when these species are exposed to a single stressor, they are able to effectively cope without altering normal body functions [38]. However, when these animals are exposed to two or more stressors simultaneously, the combined stress has a negative influence on growth [37,38] and reproduction [34,36]. This is associated with the animal’s inability to cope with cumulative effects of multiple stressors. In these instances, the animal’s body reserves are not sufficient to effectively counter exposure to these stressors. As a result, the adaptive capability of the animals is reduced, and there is an inability to maintain normal homeothermy [32,35]. 

**Figure 1:**
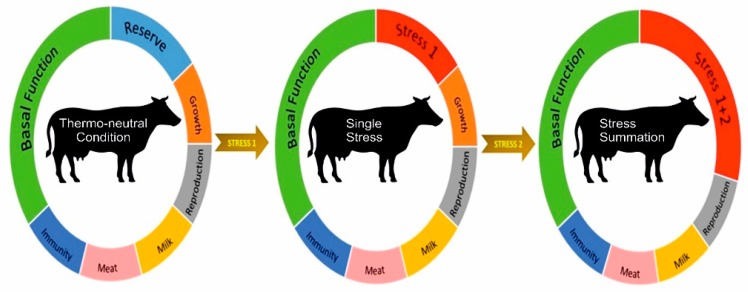
Schematic highlighting the concept of multiple stressors on cattle (adopted and modified from Sejian et al. [30]).

Although the concept of multiple stressors is becoming a focal research topic in small ruminants, the impact of multiples stressors has not been adequately researched, and as such, there is no information on large ruminants. Therefore, it is essential to explore the impact of multiple stressors on both dairy and beef cattle, particularly in conjunction with the changing global environment. Figure 1 depicts the proposed hypothetical model describing the concept of multiple stressors in cattle. The generation of baseline information is vital as this will allow for the development of appropriate amelioration and adaptive strategies to support livestock production systems. 

## 5. Implications of Hot Environmental Conditions

Animal responses to environmental stressors have been investigated for some time, and although knowledge continues to be developed, managing livestock to reduce the negative impact of hot weather remains challenging [18,20]. Reductions in dry matter intake (DMI), growth, feed conversion efficiency [25,39,40], reproduction [41], milk production and milk quality [42,43], are commonly observed when cattle are exposed to thermal stress. Quantifiable measures, such as physiological, behavioral, and biological responses to heat load have been identified as indicators of heat load. Physiological responses to heat load include increased sweating rate [14], respiration rate, breaths per minute [44], panting score [45], and body temperature [46]. Behavioral responses include alterations to posture, including increasing the proportion of time standing, increased duration in shaded areas or increased shade seeking, including shade provided from other animals, and body splashing at water troughs [47]. Biological markers in the blood are also indicators in determining the level of stress an animal is under [48]. Cattle also use adaptive behaviors to reduce heat load, primarily consisting of shade seeking, under shade structures or other animals, and the alignment of the body in accordance with solar radiation (W/m^2^) to reduce whole-body exposure to direct sunlight [49]. 

### 5.1. Nutrition and Eating Behavior

Heat production has a positive relationship with feed intake in ruminants, and it has been shown that heat production is closely associated with feeding time [50]. Metabolic heat produced during microbial fermentation [51], accounts for 3 to 8% of the total heat production by cattle [52]. As ambient heat load increases and DMI decreases there is a reduction in metabolic heat production [50]. During hot weather, cattle compensate for the hotter conditions by consuming smaller meals, more frequently, and shifting feed intake to cooler parts of the day [40,53,54]. Voluntary feed intake has been reported to commence declining when ambient temperature reaches approximately 25 °C to 27 °C [55]. However, the ambient temperature at which DMI begins to decline is influenced by diet type and composition specifically diets with a greater proportion of roughage exhibit more rapid reductions in DMI [55]. Variations in DMI are also influenced by breed (genotype), production status, health status, body condition, and days on feed.

### 5.2. Water Intake

Water is available to animals in three forms, free drinking water, water in feed, and water produced via oxidation of organic compounds or metabolic water [56]. Water requirements of cattle are influenced by ambient conditions, diet type, breed (genotype), weight, and physiological functions [57]. Daily water intake is also influenced by a number of body functions, including the regulation of core body temperature, growth and development, lactation and reproductive functions, digestion and metabolism, and hydrolysis of proteins, fats and carbohydrates [58]. Water intake is linked to DMI, with both feed intake and feed type influencing water intake [59]. Furthermore, water intake is influenced by the amount of water gained from drinking, eating, via metabolic water, and the amount of water lost per unit time through respiration, sweating, faces, urine, and lactation [60]. Arias and Mader [57] reported that feedlot cattle finished in the summer consumed 87.3% more (*p* < 0.01) water compared to cattle finished during winter (32.4 L/d versus 17.3 L/d). Increased water consumption during summer can be attributed to increases in urine volume (25%), respiratory tract evaporation (54%), and evaporative heat loss, mainly due to sweating (177%) [59]. However, an increase in water intake may also be a reflection of ruminants attempting to compensate for heat loads, particularly in un-shaded grazing systems [61]. 

### 5.3. Metabolic Dysfunction

Digestion and absorption processes carried out by the animal are affected by the thermal environment. Primarily, during heat load, absorbable nutrients are diverted from growth and development and directed to maintaining homeostasis [62]. High heat load conditions are also associated with a reduction in gut motility and rumination [55]. When cattle start to accumulate body heat, i.e., core body temperature is increasing, there is a redistribution of blood flow from the internal organs to the extremities [63], thus away from the gastrointestinal tract, or more specifically reduced blood flow to the mucosa of the dorsal rumen (32%) and reticulum (31%) [64]. Given that there is a reduction in DMI and blood flow to the gastrointestinal tract during heat load, the concentration of absorbable nutrients per unit of blood volume must increase if the animal is to satisfy daily requirements [55] and maintain normal bodily functions. 

Additionally, heat load has been associated with a 7% to 25% increase in maintenance energy requirements [65], which is associated with energy costs required to dissipate accumulated heat load [63], e.g., via increased respiration rate. However, the increase in maintenance energy requirements does not adequately describe the total increase in energy requirements as it does not include the energy costs associated with protein synthesis or hematological responses that occur outside normal homeostasis [66,67]. Therefore, a voluntary reduction in DMI is not beneficial to animal performance and wellbeing. However, the reduction in DMI is an important contributing factor to the maintenance of core body temperature. Additionally, the effect of heat load on digestion and nutrient partitioning cannot be completely explained by the reduction in DMI [43,68]. Therefore, these metabolic changes can potentially become classified as a part of the acclimation and adaptation to hot environments, where many of the changes in metabolic pathways are not yet defined and/or understood. 

### 5.4. Body Temperature

During periods of hot weather, an increase in core body temperature becomes a function of heat accumulated and dissipated between the animal and the environment [69]. Therefore changes in body temperature can be considered to be a reliable indicator of heat storage and disrupted homeostasis [70,71]. However, it is important to consider that body temperature is not static and exhibits a circadian rhythm [72,73], although is generally regulated within a ± 1 °C gradient [46].

Under thermoneutral conditions, the core body temperature of cattle is between 38 °C to 38.5 °C [74] and a rectal temperature greater than 42 °C is considered to be lethal [75]. Verwoerd et al. [76] concluded that cattle were able to isolate their body temperature from the thermal environment during moderate temperatures, however, when conditions become hot cattle are no longer able to cope with increasing ambient conditions. Furthermore, Spiers et al. [77] indicated that rectal temperature of cattle increased within 24 h after the onset of acute heat stress. 

Under moderate conditions (18 ± 7 °C) the diurnal rhythm of body temperature has been suggested to lag ambient conditions by 8 to 10 h, i.e., body temperature will peak 8 to 10 h after the ambient temperature has peaked [24]. However, during heat wave events (32 ± 7 °C), the lag between body temperature and ambient temperature decreases to 3 to 5 h [24]. This suggests that hot conditions impede an animal’s capacity to remain in thermal equilibrium with its environment. This emphasizes that when conditions exceed the thermoneutral zone there is a breakdown in the biological mechanisms that regulate body temperature in bovines. Mehla et al., [78] indicated that as body temperature increases towards 42 °C there are numerous effects on bodily functions: (i) direct damage to cells where there is an increase in membrane fluidity and permeability, (ii) an increase in the animal’s metabolic rate, and (iii) a reduction in blood flow around the body [78]. Above 42 °C homeostatic systems within the body reach their upper critical limits for normal function [78], likely resulting in death.

### 5.5. Reproduction

Heat load has also been associated with impaired reproductive success in cattle. Some of the negative impacts on reproduction can be associated with the increase in body temperature that occurs during heat load. Declines in reproductive success are not isolated specifically to males or females during periods of heat load. This is reflected by the numerous studies that have been conducted on the impact of heat load on male and female reproduction in bovines and in other species, particularly sheep. 

#### 5.5.1. Impact on Males

Over the years, there has been an emphasis on the influence of heat load on male fertility and the role that the scrotum plays in thermoregulation of the testicles. One consistent finding across studies is that heat load, either via through scrotal insulation or whole body exposure, adversely affects spermatogenesis and/or the viability of stored spermatozoa [79,80,81,82,83,84,85,86]. Furthermore, recovery time from a single heat-related insult can be as long as eight weeks [85], however, recovery is likely to encompass a full spermatogenesis cycle [87]. There have been no studies that have reported a positive relationship between heat load and spermatogenesis. With the consequences of climate change including predictions of more extreme weather events including heat waves as well as longer and hotter summers, there is going to be the potential for increased incidences of heat load, thus thermal insults on the scrotum. What has not yet been well defined is the ability of the scrotum to maintain the optimal temperature for spermatogenesis during periods of heat load. Recently, studies have evaluated scrotal temperature, and body temperature of Wagyu bulls, where scrotal temperatures were remotely monitored whilst bulls were placed through a series of heat load regimes [88,89]. The findings from these studies highlight that the mechanisms thought to maintain scrotal temperature start to breakdown during periods of heat load [88,89]. 

#### 5.5.2. Impact on Females

Heat load impairs numerous functions associated with establishing and maintaining pregnancy, including altered follicular development and dominance patterns [90,91,92], corpus luteum regression [90], impaired ovarian function [93], impaired oocyte quality and competence [94,95,96], embryonic development [97,98], increased embryonic mortality and early fetal loss [99,100], endometrial function [101], reduced uterine blood flow [102], and reduced expression of estrus and estrus behaviors, i.e., mounting [91,95,103]. The impact of heat load on female reproduction may be more pronounced in *Bos taurus* cows, however, this does not mean that there are no negative implications for *Bos indicus* cows [95].

As heat load intensity increases there is a continuous decline in conception rates in lactating cows [91,104]. Conception rates can be influenced by a heat load event during the month preceding breeding to two weeks following breeding [105]. Heat load is also associated with smaller conceptus size, which may influence maternal recognition of pregnancy and maintenance of corpus lutea function [99]. Furthermore, heat load has been associated with compromising gestation during the peri-implantation period, where there is an increased risk in early fetal loss between days 21 to 30 of gestation [94]. This may be further confounded by a reduction in uterine blood flow, which may also influence the availability of nutrients and hormones to the uterus [102]. However, as embryonic development progresses, there is an increase in embryonic thermotolerance [97]. In conjunction with climate change, it is probable that the impact of hot weather on reproduction may become more pronounced. It has been suggested that some of the negative effects of heat load may be negated via the use of mitigation techniques [106], however, Al-Katanani et al. [96] suggest that cooling cows for 42 days did not alleviate the impact of heat load on oocyte competence. 

### 5.6. Health

Hot weather has a negative influence on animal bioenergetics, and as such has a negative influence on animal performance, health, and well-being [40,107]. Heat load has been associated with an increased incidence of nutrient deficiencies, respiratory alkalosis, ketosis, and ruminal acidosis [108]. Furthermore, in lactating dairy cows, heat load has been associated with an increased frequency and incidence of clinical mastitis [109,110]. The health status of an animal is also likely to have a significant influence on the animal’s ability to cope with heat load conditions. A study by Brown-Brandl et al. [26] reported that animals with previous treatment history for pneumonia, anytime from birth to slaughter, had respiration rates that were on average 10.5% higher compared to those never diagnosed or treated. Similarly, previous and active health ailments have been reported to decrease average daily gains in feedlot cattle [26,111]. The net effect of illness related fever and exposure to heat load conditions could potentially result in an increased risk of mortality [112]. Animal health is also likely to be impacted by disease-causing agents, including vectors and parasites that flourish during summer when the conditions are hot and humid [108].

### 5.7. Productivity

During periods of high heat load, absorbable nutrients are diverted from growth and development and directed towards maintaining body temperature [62,113]. 

#### 5.7.1. Growth

Periods of heat stress are associated with reductions in growth, i.e., live weight gains [114] and DMI [40,55]. As ambient heat load increases, cattle divert energy that is typically partitioned for growth towards maintaining homeostasis [71,108], resulting in a reduction in growth and growth efficiency. For feedlot cattle, this diversion of energy is associated with depressed growth rates, whereby heat-related decreases in weight gain are approximately 10 kg, which coincides with a seven-day increase in days on feed [62]. There is considerable variability in average daily gains and feed conversion across feedlot studies [31,115,116,117,118,119,120,121]. However, it is probable that these are reflective of weather conditions and cattle management throughout these studies. Overall reduced growth rate increases days on feed, thereby increasing the cost of production.

#### 5.7.2. Milk Production and Composition

It is widely accepted that milk yields decline during hot weather [108,122,123,124,125]. Ambient temperatures of 29 °C have been reported to reduce milk yield of dairy cows by 23% [77]. Additionally, it has been estimated that the energy requirement of the cow is 20% greater at 35 °C when compared with the energy requirements at 20 °C [126]. Reductions in milk yield during heat load are predominantly associated with reduced DMI [127]. However, only 35% to 50% of the reduction in milk yield can be accounted for via the decrease in DMI [43,68]. Heat load is considered to have a greater impact on high production cows [42,126]. This is not unexpected given the positive correlation between increased milk yield, feed intake and metabolic heat production [108]. Purwanto et al. [128] concluded that cows with milk yields of 18.5 kg/d and 31.6 kg/d had 27.3% and 48.5% greater metabolic heat production (kJ/kgW^0.75^ per h) when compared to dry cows. Another important consideration is that the impact of heat stress conditions may have prolonged effects. A reduced milk yield may be seen well after the heat load period has abated. Milk production may not return to pre-exposure production levels as the energy deficit experienced combined with a decline in body condition score cannot be compensated for, particularly in the high producing cow, resulting in a permanent reduction in milk production for the remainder of that lactation [127]. This reduction in milk yield is directly proportional to the length and severity of the heat load experienced and how adversely individual cows were impacted by the heat load [127]. 

Heat load also has a negative association with milk fat and protein composition [129]. Climatic conditions appear to have the most influence on milk composition during the first 60 days of lactation [123,130]. Furthermore, the stage of lactation, diet type and composition, health status of the cow, cow genetics, and climatic conditions are all drivers of variation in milk protein [129,131]. Protein composition is further influenced by the protein secretion of the individual cow [132]. However, numerous authors have reported a negative relationship between heat load and milk fat [125,129,130,133,134,135,136,137,138,139] and protein composition [125,129,135,136,138,139,140]. Garner et al. [139] found that cows exposed to heat produced milk with a lactose and protein composition 49% lower than thermoneutral control cows. These findings suggest that milk fat and protein composition is variable, a portion of this variability can be contributed to climatic conditions. However, it is important to consider that variations in milk composition are also related to genetic and nutritional factors [141,142].

#### 5.7.3. Dark Cutting Beef

To date, there have been limited studies investigating the influence of hot and cold conditions on carcass characteristics, meat quality or consumer acceptance. Anecdotally, Australian feedlots have reported an increased incidence of “dark cutting” during the summer months, attributing this increased incidence to heat load. Dark cutting, is a complex multifactorial problem that is influenced by numerous pre-slaughter stress factors. Dark cutting is generally attributed to low muscle glycogen stores at slaughter, which is predominantly a function of glycogenesis [143]. Muscle glycogen depletion has been associated with numerous factors including, but not limited, to nutritional status, particularly in grazing systems [144,145], water supply and quality [143], animal temperament [145,146], sex [145,146], climatic conditions and climatic variability [147], and hormone growth promotants, however, this may be confounded by sex [148]. Furthermore, periods of heat load are associated with a decrease in feed intake [40,50,55,149]. This reduction in feed intake and whole-body exposure to stressors which may result in lower muscle glycogen. Managing muscle glycogen is crucial to minimizing the incidence of dark cutting beef. Further studies are required to examine the relationship between carcass attributes and climatic conditions in cattle. Furthermore, the influence of environmental conditions and/or time of exposure to these conditions on the incidence of dark cutting is yet to be established.

## 6. Mitigation Opportunities

The provision of alleviation strategies is paramount in supporting the animals to achieve comfort and production goals. Heat load alleviation strategies are focused on reducing the impact of the thermal environment and facilitate the ability to maintain normal body temperature [150] and ultimately homeostasis. The use of cooling mechanisms is encouraged and reduces the impact of environmental conditions on productive performance [122]. Heat loss is achieved through conduction, convection, and radiation. However, all of these mechanisms are dependent on a thermal gradient [42]. As ambient temperature increases there is a shift in the cooling mechanisms utilized by animals, i.e., transitioning from non-evaporative cooling to evaporative heat loss [42].

Traditionally, strategies for mitigating of heat load have involved environmental modification where the focus has been on (i) reducing solar radiation and (ii) increasing air movement [39]. However, there have also been studies investigating wetting cattle [151]. A study by Gaughan et al. [152] investigated the influence of day and night cooling, through the use of water application and air movement, on managing heat load as determined by changes in rectal temperature, respiration rate, and DMI. Gaughan et al. [152] concluded that actively cooling cattle after maximum ambient temperature occurred, was more effective at cooling cattle when compared to animals that were cooled when ambient temperature was at its peak. Cattle that were cooled during peak ambient temperature have been suddenly exposed to hot conditions, resulting in a rapid accumulation of body heat as these cattle had not been required to initiate normal physiological responses to cope with heat load whilst being actively cooled [151].

Whilst not covered in substantial detail here, the implementation of mitigation strategies will become increasingly important in livestock production systems. There are numerous mitigation opportunities available to producers, however, here an emphasis has been placed on (i) shade structures, (ii) nutritional management, and (iii) genetics and genomic selection. Shade structures are predominantly implemented in commercial industries globally, as they are cost effective and relatively simplistic to implement. Nutritional strategies are becoming more prominent in research, particularly in light of antibiotic resistance. Whilst it is well understood that genetics has an integral role in thermotolerance, the genomic selection of livestock for heat tolerance is an emerging field of study. Mitigation opportunities need to be evaluated for individual livestock systems to ensure that the alleviation strategies implemented become an effective management tool for reducing the impact of heat load in that particular enterprise.

### 6.1. Shade Structures

It has been well established that the provision of shade is an advantageous heat load alleviation tool for lactating dairy cows [28,114,151,153,154,155,156,157,158,159,160]. The provision of shade structures reduces exposure to direct solar radiation. However, shade structures do not alter ambient temperature or relative humidity [28,159,161]. Shaded areas can reduce the radiant heat load of an animal by 30%, by simply blocking out the sun [162]. Roman-Ponce et al. [163] showed that providing shade reduced black globe temperature by approximately 8 °C. Therefore, providing shade for cattle presents a cooler microclimate that cattle can utilize to seek relief from hot weather [114]. However, the beneficial aspects of shade structures, i.e., reduced exposure to solar radiation, may be offset by a lack of air movement under the structure itself [161].

The benefits associated with the use of shade structures during hot ambient conditions have been of interest for many years [40]. The advantage of shade structures is that the application is passive, where animals are able to utilize shaded areas voluntarily [39]. Schütz et al. [153] suggested that cows preferred shade on days where ambient temperatures were ≥30 °C. The authors also noted that shade utilization was reduced when relative humidity was ≥55% [153]. Furthermore, Schütz et al. [155] reported that cows preferred shade that blocked out a higher proportion of solar radiation.

What remains clear is that as heat load increases, shade seeking behaviors also increase [155]. Entwistle et al. [22] reported that during a heat wave shade reduced the impact of severe conditions on excessive heat load related deaths, whereas unshaded pens had a higher, 5.8%, mortality rate compared with shaded pens, 0.2%. Schütz et al. [156] described that as heat stress conditions intensify there is an increase in competition for shade between cows. However, there is also some conjecture regarding the amount of shade, m^2^/animal, required to offset the impact of heat load.

### 6.2. Nutrition

Nutritional management strategies for cattle during hot conditions are focused on using (i) high energy diets [152,164], (ii) feed additives such as betaine [165,166,167], probiotic yeast supplements [168,169,170,171], and antioxidants [172], (iii) managing the proportion of roughage in the diet [173] and (iv) altering feeding time to reduce metabolic heat loads during the hottest hours of the day [174]. However, there is considerable variability in the success of these techniques during heat load. Further studies are required to ensure the appropriateness of nutritional supplements as a heat load mitigation tool. 

### 6.3. Genetics

An animal’s genotype is a major factor contributing to its susceptibility or tolerance to heat load. It is widely acknowledged that *Bos indicus* breeds have greater heat tolerance compared to *Bos taurus* breeds. Gaughan et al. [29] indicated that the identification of heat tolerant cattle is not a new concept, as many breeds are already known for their thermal tolerance, i.e., Brahman and other *Bos indicus* breeds [25]. Additionally, there are *Bos taurus* genotypes that are considered tropically adapted and able to cope with hot weather. However, it is important to consider that the ability of heat tolerant *Bos taurus* genotypes to cope with hot weather does not compare to animals of *Bos indicus* heritage [175].

Further consideration needs to be extended to the selection of breeding animals. Performance-based selection of livestock has been used for numerous decades, i.e., selection of breeding stock based on the phenotypic performance of economically important traits such as high growth rates. In future years, producers will continue to select replacement breeding stock based on individual performances for traits that are deemed economically important. Rhoades et al. [176] suggested that whilst genetic improvement programs continue to place emphasis on these economically important traits, there is the potential that this will decrease thermotolerance due to the relationship that is observed between animal productivity and increasing metabolic heat production. This increase in metabolic heat production typically reduces the thermoneutral zone of these animals, and in conjunction with climate change may present some difficulty in managing cattle during hot weather. 

### 6.4. Genomic Selection for Heat Tolerance

Recently, there have been studies investigating the potential for genomic selection for heat tolerance in dairy cattle [177,178,179,180]. Genomic selection for heat tolerance has the potential to have cumulative and permanent effects [178], on heat tolerance in production species. Whilst research in this area continues to develop, the commercial viability of selection for heat tolerance needs to be evaluated. It is also important to consider that the selection for one trait may have negative consequences for another trait. It is generally accepted that improved heat tolerance comes at the cost of growth and reproduction [29]. However, there remains some conjecture regarding this, Sánchez et al. [181] suggested that cows with higher heat tolerance would have a lower rate of decline in production, although cows that are considered as ‘low production’ cows do not exhibit as severe declines in production [182], therefore may be classified as thermotolerant. However, it is more likely that this thermotolerance is related to the proportion of heat dissipation required by high production cows. It is known that high production cows produce a greater proportion of metabolic heat. Cows with milk yields of 18.5 kg/d and 31.6 kg/d had 27.3% and 48.5% greater metabolic heat production (kJ/kgW^0.75^ per h) when compared to dry cows [128]. Thus, high producing cows may be more susceptible to hot weather, regardless of genomic selection. Furthermore, it is unclear if declines in milk production provide the ‘best’ evaluation of heat tolerance in dairy cows. Other measures such as evaluation of body temperature may be a more reliable estimate of heat tolerance. Some consideration must also be extended to the impact of epigenetic mechanisms that regulate thermotolerance as well as understanding of transgenerational effects [183,184]. Recently, there have been studies attempting to quantify epigenetic change in cattle populations [184,185,186,187,188].

## 7. Adaptation and Acclimation

It is important to consider that all animals possess the capacity to adapt to their thermal environment. Animals are capable of modifying their behavioral, physiological, and morphological, or a combination of these, characteristics in response to the thermal environment [7]. Thus all animals have developed survival techniques that minimize the effect that heat load has on the body as a whole. The coping mechanisms developed by animals can be summarized into adaptation and acclimation. Adaptation and acclimation have different meanings, however, they are often interchanged [21].

### 7.1. Acclimation

Acclimation is a homeostatic process that is driven by the endocrine system, resulting in cellular, metabolic, and systemic changes, enabling animals to respond and cope with thermal stressors. Acclimation can be separated into (i) developmental and (ii) reversible [7]. Developmental acclimation refers to irreversible changes, and reversible acclimation refers to regulated animal responses, i.e., changes in response to the changing seasons [7], such as changing coat characteristics. Therefore, acclimation can be considered as a within a lifetime process whereby continuous exposure to a particular stressor, i.e., hot weather, results in biological adjustments thereby increasing the fitness of that individual animal to survive in those conditions [189]. Horowitz [189] also indicated that a part of the acclimation response is a widening in the dynamic range of body temperature, resulting in greater shifts in upper and lower critical temperature. Hahn and Mader [24] reported that cattle appear to be acclimating when post heat wave body temperature transitioned and stabilized around a new elevated temperature. Changing the dynamic range in body temperature will have a positive influence on the regulation of body temperature through adjustments to heat accumulation and dissipation from the body. 

### 7.2. Adaptation

Adaptation refers to the biological change in successive generations by favoring genetic selection within a population due to continuous stressor exposure that supports species survival [190]. *Bos indicus* cattle evolved in tropical regions, with high ambient temperature and relative humidity and as a result, these breeds of cattle have a number of genetic differences that support thermotolerance [190,191]. Therefore, the survivability of *Bos indicus* breeds in tropical environments arises from the adaptations developed throughout successive generations. In grazing breeding herds there is the potential that climate change will be a driver for the ‘natural’ selection for heat tolerant cattle, regardless of selection pressures placed on the population. The adaptation of successive generations has the potential to enhance the progeny’s ability to cope with hot conditions, although this is somewhat difficult to define in bovines due to long generation intervals. When acclimation and adaptation occur, they provide a level of resilience within cattle populations. Furthermore, in conjunction with the use of mitigation opportunities, acclimation and adaptation have the potential to enhance cattle welfare and productivity during periods of heat load. 

## 8. Conclusions

Climatic conditions are an important regulator in agricultural production systems worldwide. For livestock production, climate change has the potential to alter the thermal environment, which may have a negative impact on welfare and productivity. It is clearly evident that the thermal environment has an influence on the wellbeing and productivity of bovines. Regardless of climate change and the predicted changes to the thermal environment, hot weather will continue to incite heat load responses in cattle worldwide. Therefore, it is imperative that livestock production systems identify and utilize mitigation strategies that are efficient and effective at reducing heat load. In future years, an integrated approach to the adoption and management of mitigation opportunities will become increasingly important to support the sustainability of livestock production systems.

In anticipation of climate change and climate variability, there is a need to develop a greater understanding of the impact global warming is likely to have on biological parameters in cattle [12]. However, this may be somewhat misleading as there is a level of uncertainty in the climate change predictions and what effect the changes will have on livestock in the coming decades. A more achievable objective may be to identify and establish effective management strategies for livestock under suboptimal conditions, rather than selection for maximum productivity and/or adaptability [8]. Furthermore, there is a need to accurately quantify the indirect effects of climate change on livestock enterprises, such as changing soil quality, water availability, grain, and pasture resources, and the changing distribution of diseases and pathogens [9,12]. Developing a comprehensive understanding of the factors that influence heat load, including climatic, environmental, and animal, will allow for innovative mitigation strategies to be established. Enhancing mitigation strategies provides an opportunity for the continual improvement of animal welfare and productivity during periods of heat load. 

## References

[1] Ames D. (1980). Thermal Environment Affects Production Efficiency of Livestock. BioScience.

[2] Mader T.L., Griffin D. (2015). Management of Cattle Exposed to Adverse Environmental Conditions. Vet. Clin. N. Am. Food Anim. Pract..

[3] Belasco E.J., Cheng Y., Schroeder T.C. (2015). The impact of extreme weather on cattle feeding profits. J. Agric. Resour. Econ..

[4] St-Pierre N.R., Cobanov B., Schnitkey G. (2003). Economic Losses from Heat Stress by US Livestock Industries. J. Dairy Sci..

[5] Sackett D., Holmes P., Abbot K., Jephcott S., Barber M. (2006). Assessing the Economic Cost of Endemic Disease on the Profitability of Australian Beef Cattle and Sheep Producers.

[6] Sejian V., Bhatta R., Soren N.M., Malik P.K., Ravindra J.P., Prasad C., Lal R., Sejian V., Gaughan J., Baumgard L., Prasad C. (2015). Introduction to Concepts of Climate Change Impact on Livestock and Its Adaptation and Mitigation. Climate Change Impact on Livestock: Adaptation and Mitigation.

[7] Angilletta M.J. (2009). Thermal Acclimation. Thermal Adaptation: A Theoretical and Empirical Synthesis.

[8] Gaughan J., Cawdell-Smith A.J., Sejian V., Gaughan J., Baumgard L., Prasad C. (2015). Impact of Climate Change on Livestock Production and Reproduction. Climate Change Impact on Livestock: Adaptation and Mitigation.

[9] Nardone A., Ronchi B., Lacetera N., Ranieri M.S., Bernabucci U. (2010). Effects of climate changes on animal production and sustainability of livestock systems. Livest. Sci..

[10] Nidumolu U., Crimp S., Gobbett D., Laing A., Howden M., Little S. (2014). Spatio-temporal modelling of heat stress and climate change implications for the Murray dairy region, Australia. Int. J. Biometeorol..

[11] Hennessey K., Fitzharris B., Bates B.C., Harvey N., Howden S.M., Hughes L., Salinger J., Warrick R., Parry M.L., Canziani O.F., Palutikof J.P., van der Linden P.J., Hanson C.E. (2007). Australia and New Zealand. Climate Change 2007: Impacts, Adaptation, Vulnerability. Contribution of Working Group II to the Fourth Assessment Report of the Intergovernmental Panel on Climate Change.

[12] Henry B., Charmley E., Eckard R., Gaughan J.B., Hegarty R. (2012). Livestock production in a changing climate: Adaptation and mitigation research in Australia. Crop Pasture Sci..

[13] Nienaber J.A., Hahn G.L., Brown-Brandl T.M., Eigenberg R.A. Summer Heat Waves—Extreme Years. Proceedings of the ASABE Annual International Meeting.

[14] Mader T.L., Gaughan J.B., Johnson L.J., Hahn G.L. (2010). Tympanic temperature in confined beef cattle exposed to excessive heat load. Int. J. Biometeorol..

[15] Solomon S., Qin D., Manning M., Marquis M., Averyt K., Tignore M.M.B. (2007). Climate Change 2007: The Physical Science Basis.

[16] Westcott N.E. (2011). The Prolonged 1954 Midwestern, U.S. Heat Wave: Impacts and Responses. Weather. Clim. Soc..

[17] Robinson P.J. (2001). On the Definition of a Heat Wave. J. Appl. Meteorol..

[18] Mader T.L. (2003). Environmental stress in confined beef cattle. J. Anim. Sci..

[19] Blackshaw J., Blackshaw A. (1994). Heat stress in cattle and the effect of shade on production and behaviour: A review. Aust. J. Exp. Agric..

[20] Hahn G.L. (1999). Dynamic Responses of Cattle to Thermal Heat Loads. J. Anim. Sci..

[21] Gaughan J.B. (2002). Respiration Rate and Rectal Temperature Responses of Feedlot Cattle in Dynamic, Thermally Challenging Environments.

[22] Entwistle K., Rose M., McKiernan B. (2000). Mortalities in Feedlot Cattle at Prime City Feedlot, Tabbita, NSW, February 2000.

[23] Bushby D., Loy D. (1997). Heat Stress in Feedlot Cattle: Producer Survey Results.

[24] Hahn G.L., Mader T.L., Bottcher R.W., Hoff S.J. (1997). Heat Waves in Relation to Thermoregulation, Feeding Behaviour and Mortality of Feedlot Cattle. Livestock Environment V, Proceedings of the Fifth International Symposium.

[25] Brown-Brandl T.M., Nienaber J.A., Eigenberg R.A., Mader T.L., Morrow J.L., Dailey J.W. (2006). Comparison of heat tolerance of feedlot heifers of different breeds. Livest. Sci..

[26] Brown-Brandl T.M., Eigenberg R.A., Nienaber J.A. (2006). Heat stress risk factors of feedlot heifers. Livest. Sci..

[27] Associated Press Thousands of Cows Die in California Heat Wave; Disposing Them Becomes a Problem. https://www.latimes.com/local/lanow/la-me-cattle-deaths-20170708-story.html.

[28] Buffington D., Collazo-Arocho A., Canton G., Pitt D., Thatcher W., Collier R. (1981). Black Globe-Humidity Index (BGHI) as a Comfort Equation for Dairy Cows. Trans. Am. Soc. Agric. Eng..

[29] Gaughan J.B., Mader T.L., Holt S.M., Sullivan M.L., Hahn G.L. (2010). Assessing the heat tolerance of 17 beef cattle genotypes. Int. J. Biometeorol..

[30] Sejian V., Bhatta R., Gaughan J.B., Dunshea F.R., Lacetera N. (2018). Review: Adaptation of animals to heat stress. Animal.

[31] Gaughan J., Kreikemeier W., Mader T. (2005). Hormonal growth-promotant effects on grain-fed cattle maintained under different environments. Int. J. Biometeorol..

[32] Sejian V., Kumar D., Gaughan J.B., Naqvi S.M.K. (2017). Effect of multiple environmental stressors on the adaptive capability of Malpura rams based on physiological responses in a semi-arid tropical environment. J. Vet. Behav. Clin. Appl. Res..

[33] Sejian V., Maurya V.P., Naqvi S.M. (2012). Effect of walking stress on growth, physiological adaptability and endocrine responses in Malpura ewes in a semi-arid tropical environment. Int. J. Biometeorol..

[34] Sejian V., Maurya V.P., Naqvi S.M.K. (2011). Effect of thermal stress, restricted feeding and combined stresses (thermal stress and restricted feeding) on growth and plasma reproductive hormone levels of Malpura ewes under semi-arid tropical environment. J. Anim. Physiol. Anim. Nutr..

[35] Shilja S., Sejian V., Bagath M., Mech A., David C., Kurien E., Varma G., Bhatta R. (2016). Adaptive capability as indicated by behavioral and physiological responses, plasma HSP70 level, and PBMC HSP70 mRNA expression in Osmanabadi goats subjected to combined (heat and nutritional) stressors. Int. J. Biometeorol..

[36] Kumar D., Sejian V., Gaughan J.B., Naqvi S.M.K. (2017). Biological functions as affected by summer season-related multiple environmental stressors (heat, nutritional and walking stress) in Malpura rams under semi-arid tropical environment. Biol. Rhythm Res..

[37] Abdul Niyas P., Sejian V., Bagath M., Parthipan S., Selvaraju S., Manjunathareddy G., Kurien E., Varma G., Bhatta R. (2017). Effect of heat and nutritional stress on growth and testicular HSP70 expression in goats. J. Agrometeorol..

[38] Sejian V., Maurya V.P., Kumar K., Naqvi S.M.K. (2012). Effect of multiple stresses on growth and adaptive capability of Malpura ewes under semi-arid tropical environment. Trop. Anim. Health Prod..

[39] Eigenberg R.A., Brown-Brandl T.M., Nienaber J.A., Hahn G.L. (2005). Dynamic Response Indicators of Heat Stress in Shaded and Non-shaded Feedlot Cattle, Part 2: Predictive Relationships. Biosyst. Eng..

[40] Brown-Brandl T.M., Eigenberg R.A., Nienaber J.A., Hahn G.L. (2005). Dynamic Response Indicators of Heat Stress in Shaded and Non-shaded Feedlot Cattle, Part 1: Analyses of Indicators. Biosyst. Eng..

[41] Jordan E.R. (2003). Effects of Heat Stress on Reproduction. J. Dairy Sci..

[42] West J.W. (2003). Effects of Heat-Stress on Production in Dairy Cattle. J. Dairy Sci..

[43] Rhoads M.L., Rhoads R.P., VanBaale M.J., Collier R.J., Sanders S.R., Weber W.J., Crooker B.A., Baumgard L.H. (2009). Effects of heat stress and plane of nutrition on lactating Holstein cows: I. Production, metabolism, and aspects of circulating somatotropin. J. Dairy Sci..

[44] Gaughan J.B., Holt S.M., Hahn G.L., Mader T.L., Eigenberg R.A. (2000). Respiration Rate—Is It a Good Measure of Heat Stress in Cattle. Asian-Australas J. Anim. Sci..

[45] Mader T.L., Davis M.S., Brown-Brandl T.M. (2006). Environmental factors influencing heat stress in feedlot cattle. J. Anim. Sci..

[46] Robertshaw D., Yousef M.K. (1985). Heat Loss of Cattle. Stress Physiology in Livestock.

[47] Young B.A., Hall A.B., Coombes R. (1993). Heat load in cattle in the Australian Environment. Australian Beef.

[48] Collier R.J., Collier J.L., Rhoads R.P., Baumgard L.H. (2008). Invited Review: Genes Involved in the Bovine Heat Stress Response. J. Dairy Sci..

[49] Nienaber J.A., Hahn G.L., Brown-Brandl T.M., Eigenberg R.A. Heat stress climatic conditions and the physiological responses of cattle. Proceedings of the Fifth International Dairy Housing.

[50] Brown-Brandl T.M., Nienaber J.A., Eigenberg R.A., Hahn G.L., Freetly H. (2003). Thermoregulatory responses of feeder cattle. J. Therm. Biol..

[51] Beatty D.T., Barnes A., Taylor E., Maloney S.K. (2008). Do changes in feed intake or ambient temperature cause changes in cattle rumen temperature relative to core temperature?. J. Therm. Biol..

[52] Czerkawski J.W. (1980). A novel estimate of the magnitude of heat produced in the rumen. Br. J. Nutr..

[53] Ray D.E., Roubicek C.B. (1971). Behavior of feedlot cattle during two seasons. J. Anim. Sci..

[54] Hicks R., Owens F., Gill D. (1989). Behavioral Patterns of Feedlot Steers.

[55] Beede D.K., Collier R.J. (1986). Potential Nutritional Strategies for Intensively Managed Cattle during Thermal Stress. J. Anim. Sci..

[56] NRC (1981). Effect of Environment on Nutrient Requirements of Domestic Animals.

[57] Arias R.A., Mader T.L. (2011). Environmental factors affecting daily water intake on cattle finished in feedlots. J. Anim. Sci..

[58] NRC (2000). Nutrient Requirements of Beef Cattle.

[59] McDowell R.E., Weldy J.R. Water Exhcange of cattle under heat stress. Proceedings of the 3rd International Biometeorological Congress.

[60] Black A.L., Baker N.F., Bartley J.C., Chapman T.E., Phillips R.W. (1964). Water Turnover in Cattle. Science.

[61] Silanikove N. (1992). Effects of water scarcity and hot environment on appetite and digestion in ruminants: A review. Livest. Prod. Sci..

[62] Baumgard L.H., Rhoads R.P. (2012). RUMINANT NUTRITION SYMPOSIUM: Ruminant Production and Metabolic Responses to Heat Stress. J. Anim. Sci..

[63] Baumgard L.H., Rhoads R.P. The Effects of Hyperthermia on Nutrient Paritioning. https://www.sid.ir/En/Journal/ViewPaper.aspx?ID=352520.

[64] Engelhardt W.V., Hales J.R.S. (1977). Partition of capillary blood flow in rumen, reticulum, and omasum of sheep. Am. J. Physiol..

[65] NRC (2001). Nutrient Requirements of Beef Cattle.

[66] Carroll J.A., Burdick Sanchez N.C., Bill E. (2014). Kunkle Interdisciplinary Beef Symposium: Overlapping physiological responses and endocrine biomarkers that are indicative of stress responsiveness and immune function in beef cattle. J. Anim. Sci..

[67] Baumgard L.H., Rhoads R.P. (2013). Effects of Heat Stress on Postabsorptive Metabolism and Energetics. Annu. Rev. Anim. Biosci..

[68] Wheelock J.B., Rhoads R.P., VanBaale M.J., Sanders S.R., Baumgard L.H. (2010). Effects of heat stress on energetic metabolism in lactating Holstein cows. J. Dairy Sci..

[69] Lees A.M., Sejian V., Lees J.C., Sullivan M.L., Lisle A.T., Gaughan J.B. (2019). Evaluating rumen temperature as an estimate of core body temperature in Angus feedlot cattle during summer. Int. J. Biometeorol..

[70] Maurya V.P., Sejian V., Gupta M., Dangi S.S., Kushwaha A., Singh G., Sarkar M., Sejian V., Gaughan J., Baumgard L., Prasad C. (2015). Adaptive Mechanisms of Livestock to Changing Climate. Climate Change Impact on Livestock: Adaptation and Mitigation.

[71] Ravagnolo O., Misztal I. (2002). Effect of Heat Stress on Nonreturn Rate in Holsteins: Fixed-Model Analyses. J. Dairy Sci..

[72] Bitman J., Lefcourt A., Wood D.L., Stroud B. (1984). Circadian and Ultradian Temperature Rhythms of Lactating Dairy Cows. J. Dairy Sci..

[73] Lefcourt A.M., Huntington J.B., Akers R.M., Wood D.L., Bitman J. (1999). Circadian and ultradian rhythms of body temperature and peripheral concentrations of insulin and nitrogen in lactating dairy cows. Domest. Anim. Endocrinol..

[74] Sjaastad O.V., Hove K., Sand O. (2003). Physiology of Domestic Animals.

[75] Findlay J.D. (1958). Physiological Reactions of Cattle to Climatic Stress. Proc. Nutr. Soc..

[76] Verwoerd W., Wellby M., Barrell G. (2006). Absence of a causal relationship between environmental and body temperature in dairy cows (*Bos taurus*) under moderate climatic conditions. J. Therm. Biol..

[77] Spiers D.E., Spain J.N., Sampson J.D., Rhoads R.P. (2004). Use of physiological parameters to predict milk yield and feed intake in heat-stressed dairy cows. J. Therm. Biol..

[78] Mehla K., Magotra A., Choudhary J., Singh A.K., Mohanty A.K., Upadhyay R.C., Srinivasan S., Gupta P., Choudhary N., Antony B. (2014). Genome-wide analysis of the heat stress response in Zebu (Sahiwal) cattle. Gene.

[79] Casady R.B., Myers R.M., Legates J.E. (1953). The Effect of Exposure to High Ambient Temperature on Spermatogenesis in the Dairy Bull. J. Dairy Sci..

[80] Johnston J.E., Naelapaa H., Frye J.B. (1963). Physiological Responses of Holstein, Brown Swiss and Red Sindhi Crossbred Bulls Exposed to High Temperatures and Humidities. J. Anim. Sci..

[81] Kastelic J., Cook R.B., Coulter G.H. (1996). Contribution of the scrotum and testes to scrotal and testicular thermoregulation in bulls and rams. Reproduction.

[82] Kastelic J.P., Cook R.B., Coulter G.H., Saacke R.G. (1996). Insulating the scrotal neck affects semen quality and scrotal/testicular temperatures in the bull. Theriogenology.

[83] Meyerhoeffer D.C., Wells M.E., Wettemann R.P., Coleman S.W. (1985). Reproductive Criteria of Beef Bulls during and after Exposure to Increased Ambient Temperature. J. Anim. Sci..

[84] Meyerhoeffer D.C., Turman E.J., Minton J.E., Hintz R.L., Wettemann R.P. (1981). Serum Luteinizing Hormone and Testosterone in Bulls during Exposure to Elevated Ambient Temperature. J. Anim. Sci..

[85] Skinner J.D., Louw G.N. (1966). Heat stress and spermatogenesis in *Bos indicus* and *Bos taurus* cattle. J. Appl. Physiol..

[86] Vogler C.J., Bame J.H., DeJarnette J.M., McGilliard M.L., Saacke R.G. (1993). Effects of elevated testicular temperature on morphology characteristics of ejaculated spermatozoa in the bovine. Theriogenology.

[87] Cruz Júnior C.A., Lucci C.M., Peripolli V., Silva A.F., Menezes A.M., Morais S.R.L., Araújo M.S., Ribeiro L.M.C.S., Mattos R.C., McManus C. (2015). Effects of testicle insulation on seminal traits in rams: Preliminary study. Small Rumin. Res..

[88] Wallage A.L., Gaughan J.B., Lisle A.T., Beard L., Collins C.W., Johnston S.D. (2017). Measurement of bovine body and scrotal temperature using implanted temperature sensitive radio transmitters, data loggers and infrared thermography. Int. J. Biometeorol..

[89] Wallage A.L., Johnston S.D., Lisle A.T., Beard L., Lees A.M., Collins C.W., Gaughan J.B. (2017). Thermoregulation of the bovine scrotum 1: Measurements of free-range animals in a paddock and pen. Int. J. Biometeorol..

[90] Wilson S.J., Kirby C.J., Koenigsfeld A.T., Keisler D.H., Lucy M.C. (1998). Effects of Controlled Heat Stress on Ovarian Function of Dairy Cattle. 2. Heifers. J. Dairy Sci..

[91] Schüller L.K., Michaelis I., Heuwieser W. (2017). Impact of heat stress on estrus expression and follicle size in estrus under field conditions in dairy cows. Theriogenology.

[92] Wolfenson D., Thatcher W.W., Badinga L., Savio J.D., Meidan R., Lew B.J., Braw-tal R., Berman A. (1995). Effect of Heat Stress on Follicular Development during the Estrous Cycle in Lactating Dairy Cattle. Biol. Reprod..

[93] Jonsson N.N., McGowan M.R., McGuigan K., Davison T.M., Hussain A.M., Kafi M., Matschoss A. (1997). Relationships among calving season, heat load, energy balance and postpartum ovulation of dairy cows in a subtropical environment. Anim. Reprod. Sci..

[94] García-Ispierto I., López-Gatius F., Santolaria P., Yániz J.L., Nogareda C., López-Béjar M., De Rensis F. (2006). Relationship between heat stress during the peri-implantation period and early fetal loss in dairy cattle. Theriogenology.

[95] Torres-Júnior J.R.D.S., Pires M.D.F.A., de Sá W.F., Ferreira A.D.M., Viana J.H.M., Camargo L.S.A., Ramos A.A., Folhadella I.M., Polisseni J., de Freitas C. (2008). Effect of maternal heat-stress on follicular growth and oocyte competence in *Bos indicus* cattle. Theriogenology.

[96] Al-Katanani Y.M., Paula-Lopes F.F., Hansen P.J. (2002). Effect of Season and Exposure to Heat Stress on Oocyte Competence in Holstein Cows. J. Dairy Sci..

[97] Ealy A.D., Aréchiga C.F., Howell J.L., Hansen P.J., Monterroso V.H. (1995). Developmental changes in sensitivity of bovine embryos to heat shock and use of antioxidants as thermoprotectants2. J. Anim. Sci..

[98] Gendelman M., Aroyo A., Yavin S., Roth Z. (2010). Seasonal effects on gene expression, cleavage timing, and developmental competence of bovine preimplantation embryos. Reproduction.

[99] Biggers B.G., Buchanan D.S., Geisert R.D., Wetteman R.P. (1987). Effect of Heat Stress on Early Embryonic Development in the Beef Cow. J. Anim. Sci..

[100] Ryan D.P., Blakewood E.G., Munyakazi L., Godke R.A., Lynn J.W. (1992). Effect of heat-stress on bovine embryo development in vitro. J. Anim. Sci..

[101] Wolfenson D., Roth Z., Meidan R. (2000). Impaired reproduction in heat-stressed cattle: Basic and applied aspects. Anim. Reprod. Sci..

[102] Roman-Ponce H., Thatcher W.W., Caton D., Barron D.H., Wilcox C.J. (1978). Thermal Stress Effects on Uterine Blood Flow in Dairy Cows. J. Anim. Sci..

[103] Pennington J.A., Albright J.L., Diekman M.A., Callahan C.J. (1985). Sexual Activity of Holstein Cows: Seasonal Effects. J. Dairy Sci..

[104] Schüller L.K., Burfeind O., Heuwieser W. (2014). Impact of heat stress on conception rate of dairy cows in the moderate climate considering different temperature–humidity index thresholds, periods relative to breeding, and heat load indices. Theriogenology.

[105] Morton J.M., Tranter W.P., Mayer D.G., Jonsson N.N. (2007). Effects of Environmental Heat on Conception Rates in Lactating Dairy Cows: Critical Periods of Exposure. J. Dairy Sci..

[106] Hansen P.J., Areéchiga C.F. (1999). Strategies for managing reproduction in the heat-stressed dairy cow. J. Anim. Sci..

[107] Collier R.J., Beede D.K., Thatcher W.W., Israel L.A., Wilcox C.J. (1982). Influences of Environment and Its Modification on Dairy Animal Health and Production. J. Dairy Sci..

[108] Kadzere C.T., Murphy M.R., Silanikove N., Maltz E. (2002). Heat stress in lactating dairy cows: A review. Livest. Prod. Sci..

[109] Morse D., DeLorenzo M.A., Wilcox C.J., Collier R.J., Natzke R.P., Bray D.R. (1988). Climatic Effects on Occurrence of Clinical Mastitis. J. Dairy Sci..

[110] Howell D., Wilson C.D., Vessey M.P. (1964). A survey of the incidence of mastitis in dairy cows in the Reading area. Vet. Rec..

[111] Gardner B.A., Dolezal H.G., Bryant L.K., Owens F.N., Smith R.A. (1999). Health of finishing steers: Effects on performance, carcass traits, and meat tenderness. J. Anim. Sci..

[112] Silanikove N. (2000). Effects of heat stress on the welfare of extensively managed domestic ruminants. Livest. Prod. Sci..

[113] DeShazer J.A., Hahn G.L., Xinm H., DeShazer J.A. (2009). Basic Principals of the Thermal Environment and Livestock Energetics. Livestock Energetics and Thermal Environmental Management.

[114] Mitlöhner F.M., Galyean M.L., McGlone J.J. (2002). Shade effects on performance, carcass traits, physiology, and behavior of heat-stressed feedlot heifers. J. Anim. Sci..

[115] Gaughan J.B., Mader T.L. (2014). Body temperature and respiratory dynamics in un-shaded beef cattle. Int. J. Biometeorol..

[116] Gaughan J.B., Bonner S., Loxton I., Mader T.L., Lisle A., Lawrence R. (2010). Effect of shade on body temperature and performance of feedlot steers. J. Anim. Sci..

[117] Lees A.M., Lees J.C., Lisle A.T., Sullivan M.L., Gaughan J.B. (2018). Effect of heat stress on rumen temperature of three breeds of cattle. Int. J. Biometeorol..

[118] Sullivan M.L., Cawdell-Smith A.J., Mader T.L., Gaughan J.B. (2011). Effect of shade area on performance and welfare of short-fed feedlot cattle. J. Anim. Sci..

[119] Lees A.M., Lees J.C., Sejian V., Wallage A.L., Gaughan J.B. (2018). Short communication: Using infrared thermography as an in situ measure of core body temperature in lot-fed Angus steers. Int. J. Biometeorol..

[120] Clarke M., Kelly A. (1996). Some effects of shade on Hereford steers in a feedlot. Proc. Aust. Soc. Anim. Prod..

[121] Gaughan J.B., Bonner S.L., Loxton I., Mader T.L. (2013). Effects of chronic heat stress on plasma concentration of secreted heat shock protein 70 in growing feedlot cattle. J. Anim. Sci..

[122] Avendaño-Reyes L., Álvarez-Valenzuela F.D., Correa-Calderón A., Algándar-Sandoval A., Rodríguez-González E., Pérez-Velázquez R., Macías-Cruz U., Díaz-Molina R., Robinson P.H., Fadel J.G. (2010). Comparison of three cooling management systems to reduce heat stress in lactating Holstein cows during hot and dry ambient conditions. Livest. Sci..

[123] McDowell R.E., Hooven N.W., Camoens J.K. (1976). Effect of Climate on Performance of Holsteins in First Lactation. J. Dairy Sci..

[124] TapkI I., Sahin A. (2006). Comparison of the thermoregulatory behaviours of low and high producing dairy cows in a hot environment. Appl. Anim. Behav. Sci..

[125] Bouraoui R., Lahmar M., Abdessalem M., Djemali M.n., Belyea R. (2002). The relationship of temperature-humidity index with milk production of dairy cows in a Mediterranean climate. Anim. Res..

[126] Staples C.R., Thatcher W.W., John W. (2011). Stress in Dairy Animals | Heat Stress: Effects on Milk Production and Composition. Encyclopedia of Dairy Sciences.

[127] Ravagnolo O., Misztal I. (2000). Genetic Component of Heat Stress in Dairy Cattle, Parameter Estimation. J. Dairy Sci..

[128] Purwanto B., Abo Y., Sakamoto R., Furumoto F., Yamamoto S. (1990). Diurnal patterns of heat production and heart rate under thermoneutral conditions in Holstein Friesian cows differing in milk production. J. Agric. Sci..

[129] Lambertz C., Sanker C., Gauly M. (2014). Climatic effects on milk production traits and somatic cell score in lactating Holstein-Friesian cows in different housing systems. J. Dairy Sci..

[130] Sharma A., Rodriguez L., Mekonnen G., Wilcox C., Bachman K., Collier R. (1983). Climatological and Genetic Effects on Milk Composition and Yield. J. Dairy Sci..

[131] Heck J.M.L., Schennink A., van Valenberg H.J.F., Bovenhuis H., Visker M.H.P.W., van Arendonk J.A.M., van Hooijdonk A.C.M. (2009). Effects of milk protein variants on the protein composition of bovine milk. J. Dairy Sci..

[132] Pollott G.E. (2004). Deconstructing Milk Yield and Composition During Lactation Using Biologically Based Lactation Models. J. Dairy Sci..

[133] Rodriquez L.A., Mekonnen G., Wilcox C.J., Martin F.G., Krienke W.A. (1985). Effects of Relative Humidity, Maximum and Minimum Temperature, Pregnancy, and Stage of Lactation on Milk Composition and Yield. J. Dairy Sci..

[134] Bernabucci U., Lacetera N., Baumgard L.H., Rhoads R.P., Ronchi B., Nardone A. (2010). Metabolic and hormonal acclimation to heat stress in domesticated ruminants. Animal.

[135] Quist M.A., LeBlanc S.J., Hand K.J., Lazenby D., Miglior F., Kelton D.F. (2008). Milking-to-Milking Variability for Milk Yield, Fat and Protein Percentage, and Somatic Cell Count. J. Dairy Sci..

[136] Bernabucci U., Basiricò L., Morera P., Dipasquale D., Vitali A., Piccioli Cappelli F., Calamari L. (2015). Effect of summer season on milk protein fractions in Holstein cows. J. Dairy Sci..

[137] Sharma A.K., Rodriguez L.A., Wilcox C.J., Collier R.J., Bachman K.C., Martin F.G. (1988). Interactions of Climatic Factors Affecting Milk Yield and Composition. J. Dairy Sci..

[138] Hill D.L., Wall E. (2014). Dairy cattle in a temperate climate: The effects of weather on milk yield and composition depend on management. Animal.

[139] Garner J.B., Douglas M., Williams S.R.O., Wales W.J., Marett L.C., DiGiacomo K., Leury B.J., Hayes B.J. (2017). Responses of dairy cows to short-term heat stress in controlled-climate chambers. Anim. Prod. Sci..

[140] Bernabucci U., Lacetera N., Ronchi B., Nardone A. (2002). Effects of the hot season on milk protein fractions in Holstein cows. Anim. Res..

[141] Ferris T.A., Vasavada P.C. (1989). Altering Milk Composition—An Introduction. J. Dairy Sci..

[142] Laben R.C. (1963). Factors Responsible for Variation in Milk Composition. J. Dairy Sci..

[143] Loudon K.M.W., Lean I.J., Pethick D.W., Gardner G.E., Grubb L.J., Evans A.C., McGilchrist P. (2018). On farm factors increasing dark cutting in pasture finished beef cattle. Meat Sci..

[144] McGilchrist P., Alston C.L., Gardner G.E., Thomson K.L., Pethick D.W. (2012). Beef carcasses with larger eye muscle areas, lower ossification scores and improved nutrition have a lower incidence of dark cutting. Meat Sci..

[145] Voisinet B.D., Grandin T., O’Connor S.F., Tatum J.D., Deesing M.J. (1997). *Bos indicus*-cross feedlot cattle with excitable temperaments have tougher meat and a higher incidence of borderline dark cutters. Meat Sci..

[146] Voisinet B.D., Grandin T., Tatum J.D., O’Connor S.F., Struthers J.J. (1997). Feedlot cattle with calm temperaments have higher average daily gains than cattle with excitable temperaments. J. Anim. Sci..

[147] McGilchrist P., Perovic J.L., Gardner G.E., Pethick D.W., Jose C.G. (2014). The incidence of dark cutting in southern Australian beef production systems fluctuates between months. Anim. Prod. Sci..

[148] Scanga J.A., Belk K.E., Tatum J.D., Grandin T., Smith G.C. (1998). Factors contributing to the incidence of dark cutting beef. J. Anim. Sci..

[149] Hahn G.L., Yousef M.K. (1985). Management and Housing of Farm Animals in Hot Environments. Stress Physiology in Livestock.

[150] Sanchez W.K., McGuire M.A., Beede D.K. (1994). Macromineral Nutrition by Heat Stress Interactions in Dairy Cattle: Review and Original Research. J. Dairy Sci..

[151] Gaughan J.B., Davis M.S., Mader T.L. (2004). Wetting and the physiological responses of grain-fed cattle in a heated environment. Aust. J. Agric. Res..

[152] Gaughan J.B., Mader T.L., Holt S.M. (2008). Cooling and feeding strategies to reduce heat load of grain-fed beef cattle in intensive housing. Livest. Sci..

[153] Schütz K.E., Cox N.R., Matthews L.R. (2008). How important is shade to dairy cattle? Choice between shade or lying following different levels of lying deprivation. Appl. Anim. Behav. Sci..

[154] Schütz K.E., Cox N.R., Tucker C.B. (2014). A field study of the behavioral and physiological effects of varying amounts of shade for lactating cows at pasture. J. Dairy Sci..

[155] Schütz K.E., Rogers A.R., Cox N.R., Tucker C.B. (2009). Dairy cows prefer shade that offers greater protection against solar radiation in summer: Shade use, behaviour, and body temperature. Appl. Anim. Behav. Sci..

[156] Schütz K.E., Rogers A.R., Poulouin Y.A., Cox N.R., Tucker C.B. (2010). The amount of shade influences the behavior and physiology of dairy cattle. J. Dairy Sci..

[157] Tucker C.B., Rogers A.R., Schütz K.E. (2008). Effect of solar radiation on dairy cattle behaviour, use of shade and body temperature in a pasture-based system. Appl. Anim. Behav. Sci..

[158] Gaughan J.B., Goodwin P.J., Schoorl T.A., Young B.A., Imbeah M., Mader T.L., Hall A. (1998). Shade preferences of lactating Holstein Friesian cows. Aust. J. Exp. Agric..

[159] Buffington D., Collier R., Canton G. (1983). Shade management systems to reduce heat stress for dairy cows in hot, humid climates. Trans. Am. Soc. Agric. Eng..

[160] Kendall P.E., Nielsen P.P., Webster J.R., Verkerk G.A., Littlejohn R.P., Matthews L.R. (2006). The effects of providing shade to lactating dairy cows in a temperate climate. Livest. Sci..

[161] Gaughan J.B., Tait L.A., Eigenberg R., Bryden W.L. (2004). Effect of shade on respiration rate and rectal temperature of Angus heifers. Anim. Prod. Aust..

[162] Bond T.E., Kelly C.F., Morrison S.R., Periera N. (1967). Solar, Atmospheric, and Terrestrial Radiation Received by Shaded and Unshaded Animals. Trans. Am. Soc. Agric. Eng..

[163] Roman-Ponce H., Thatcher W.W., Buffington D.E., Wilcox C.J., Van Horn H.H. (1977). Physiological and Production Responses of Dairy Cattle to a Shade Structure in a Subtropical Environment. J. Dairy Sci..

[164] Gaughan J.B., Mader T.L. (2009). Effects of sodium chloride and fat supplementation on finishing steers exposed to hot and cold conditions. J. Anim. Sci..

[165] Dunshea F.R., Oluboyede K., DiGiacomo K., Leury B.J., Cottrell J.J. (2019). Betaine Improves Milk Yield in Grazing Dairy Cows Supplemented with Concentrates at High Temperatures. Animals.

[166] DiGiacomo K., Simpson S., Leury B.J., Dunshea F.R. (2016). Dietary Betaine Impacts the Physiological Responses to Moderate Heat Conditions in a Dose Dependent Manner in Sheep. Animals.

[167] Cronje P. (2005). Heat stress in livestock–the role of the gut in its aetiology and a potential role for betaine in its alleviation. Recent Adv. Anim. Nutr. Aust..

[168] Moallem U., Lehrer H., Livshitz L., Zachut M., Yakoby S. (2009). The effects of live yeast supplementation to dairy cows during the hot season on production, feed efficiency, and digestibility. J. Dairy Sci..

[169] DeVries T.J., Chevaux E. (2014). Modification of the feeding behavior of dairy cows through live yeast supplementation. J. Dairy Sci..

[170] Crossland W.L., Cagle C.M., Sawyer J.E., Callaway T.R., Tedeschi L.O. (2019). Evaluation of active dried yeast in the diets of feedlot steers. II. Effects on rumen pH and liver health of feedlot steers. J. Anim. Sci..

[171] Crossland W.L., Jobe J.T., Ribeiro F.R.B., Sawyer J.E., Callaway T.R., Tedeschi L.O. (2019). Evaluation of active dried yeast in the diets of feedlot steers: I. Effects on feeding performance traits, the composition of growth, and carcass characteristics. J. Anim. Sci..

[172] Calamari L., Petrera F., Abeni F., Bertin G. (2011). Metabolic and hematological profiles in heat stressed lactating dairy cows fed diets supplemented with different selenium sources and doses. Livest. Sci..

[173] Mader T.L., Gaughan J.B., Young B.A. (1999). Feedlot Diet Roughage Level for Hereford Cattle Exposed to Excessive Heat Load. Prof. Anim. Sci..

[174] Brosh A., Aharoni Y., Degen A.A., Wright D., Young B.A. (1998). Effects of solar radiation, dietary energy, and time of feeding on thermoregulatory responses and energy balance in cattle in a hot environment. J. Anim. Sci..

[175] Carvalho F.A., Lammoglia M.A., Simoes M.J., Randel R.D. (1995). Breed affects thermoregulation and epithelial morphology in imported and native cattle subjected to heat stress. J. Anim. Sci..

[176] Rhoads R.P., Baumgard L.H., Suagee J.K. (2013). 2011 and 2012 Early Careers Achievement Awards: Metabolic priorities during heat stress with an emphasis on skeletal muscle. J. Anim. Sci..

[177] Nguyen T.T.T., Bowman P.J., Haile-Mariam M., Nieuwhof G.J., Hayes B.J., Pryce J.E. (2017). Short communication: Implementation of a breeding value for heat tolerance in Australian dairy cattle. J. Dairy Sci..

[178] Nguyen T.T.T., Bowman P.J., Haile-Mariam M., Pryce J.E., Hayes B.J. (2016). Genomic selection for tolerance to heat stress in Australian dairy cattle. J. Dairy Sci..

[179] Nguyen T.T.T., Hayes B.J., Pryce J.E. (2017). A practical future-scenarios selection tool to breed for heat tolerance in Australian dairy cattle. Anim. Prod. Sci..

[180] Garner J.B., Douglas M.L., Williams S.R.O., Wales W.J., Marett L.C., Nguyen T.T.T., Reich C.M., Hayes B.J. (2016). Genomic Selection Improves Heat Tolerance in Dairy Cattle. Sci. Rep..

[181] Sánchez J.P., Misztal I., Aguilar I., Zumbach B., Rekaya R. (2009). Genetic determination of the onset of heat stress on daily milk production in the US Holstein cattle. J. Dairy Sci..

[182] Lees J.C. (2018). A Heat Load Index for Dairy Cattle. Ph.D. Thesis.

[183] Singh K., Erdman R.A., Swanson K.M., Molenaar A.J., Maqbool N.J., Wheeler T.T., Arias J.A., Quinn-Walsh E.C., Stelwagen K. (2010). Epigenetic Regulation of Milk Production in Dairy Cows. J. Mammary Gland Biol. Neoplasia.

[184] Ahmed B.M.S., Younas U., Asar T.O., Dikmen S., Hansen P.J., Dahl G.E. (2017). Cows exposed to heat stress during fetal life exhibit improved thermal tolerance. J. Anim. Sci..

[185] Tao S., Dahl G.E., Laporta J., Bernard J.K., Orellana Rivas R.M., Marins T.N. (2019). Effects of heat stress during late gestation on the dam and its calf. J. Anim. Sci..

[186] Skibiel A.L., Dado-Senn B., Fabris T.F., Dahl G.E., Laporta J. (2018). In utero exposure to thermal stress has long-term effects on mammary gland microstructure and function in dairy cattle. PLoS ONE.

[187] Skibiel A.L., Peñagaricano F., Amorín R., Ahmed B.M., Dahl G.E., Laporta J. (2018). In Utero Heat Stress Alters the Offspring Epigenome. Sci. Rep..

[188] Dahl G.E., Tao S., Laporta J. (2017). TRIENNIAL LACTATION SYMPOSIUM/BOLFA: Late gestation heat stress of dairy cattle programs dam and daughter milk production. J. Anim. Sci..

[189] Horowitz M. (2001). Heat acclimation: Phenotypic plasticity and cues to the underlying molecular mechanisms. J. Therm. Biol..

[190] Roy K.S., Collier R.J., Collier R.J., Collier J.L. (2012). Regulation of Acclimation to Environmental Stress. Environmental Physiology of Livestock.

[191] Hansen P.J. (2004). Physiological and cellular adaptations of zebu cattle to thermal stress. Anim. Reprod. Sci..

